# Effects of white Gaussian noise on dynamic balance in healthy young adults

**DOI:** 10.1038/s41598-021-84706-8

**Published:** 2021-03-09

**Authors:** Ziyou Zhou, Can Wu, Zhen Hu, Yujuan Chai, Kai Chen, Tetsuya Asakawa

**Affiliations:** 1 Department of Mechanical Engineering, School of Mechanical Engineering, Hangzhou Dianzi University, No.1158, Xiasha 2nd Street, Jianggan District, Hangzhou, 310018 Zhejiang China; 2 Department of Neurology, Ruijin Hospital Affiliated to Shanghai Jiao Tong University, Shanghai, 200000 China; 3 Department of Neurosurgery, Hamamatsu University School of Medicine, Handayama, 1-20-1, Higashi-ku, Hamamatsu-City, Shizuoka 431-3192 Japan; 4 Research Base of Traditional Chinese Medicine Syndrome, Fujian University of Traditional Chinese Medicine, Fuzhou, 350122 China; 5 Health Science Center, School of Medical Engineering, Shenzhen University, Shenzhen, 518060 China

**Keywords:** Neurophysiology, Physiology, Medical research

## Abstract

It has been known that short-time auditory stimulation can contribute to the improvement of the balancing ability of the human body. The present study aims to explore the effects of white Gaussian noise (WGN) of different intensities and frequencies on dynamic balance performance in healthy young adults. A total of 20 healthy young participants were asked to stand at a dynamic balance force platform, which swung along the x-axis with an amplitude of ± 4° and frequency of 1 Hz. Their center of pressure (COP) trajectories were recorded when they were stimulated by WGN of different intensities (block 1) and different frequencies (block 2). A traditional method and detrended fluctuation analysis (DFA) were used for data preprocessing. The authors found that only with 75–85 dB WGN, the COP parameters improved. WGN frequency did not affect the dynamic balance performance of all the participants. The DFA results indicated stimulation with 75 dB WGN enhanced the short-term index and reduced the crossover point. Stimulation with 500 Hz and 2500 Hz WGN significantly enhanced the short-term index. These results suggest that 75 dB WGN and 500 Hz and 2500 Hz WGN improved the participants’ dynamic balance performance. The results of this study indicate that a certain intensity of WGN is indispensable to achieve a remarkable improvement in dynamic balance. The DFA results suggest that WGN only affected the short-term persistence, indicating the potential of WGN being considered as an adjuvant therapy in low-speed rehabilitation training.

## Introduction

Falls are a common problem in the aging population and patients with neurological deficiencies, which are closely associated with dynamic balance impairments^1^. Hence, the amelioration of the dynamic balance impairments plays a vital role in the prevention of potential falls of patients. Dynamic balance is a concept contrary to static balance that is defined as maintaining or restoring body balance as a response to internal or external disturbances^2^. Dynamic balance is the comprehensive interconnections between the visual, vestibular, somatosensory, muscular, and central nervous systems (CNS). It has been documented that short-time auditory stimulation can contribute in the improvement of the balancing ability of the human body^3,4^. The potential mechanisms lie in the fact that the auditory and vestibular feedback signals are transmitted to the brain through the acoustic nerve, which subsequently affects the temporal lobe that is directly associated with the modulation of postural balance^5^. But some authors have controversial conclusions. Park et al. reported a decline of a worker’s balance performance when exposed to noise^6^. However, the noise involved in these studies was not white Gaussian noise (WGN), and most of these studies investigated on static balance. Nevertheless, the effects of WGN on balance were also controversial. WGN has been reported to have effects to improve balance performance^7^. But Ross et al. had a converse conclusion^8^. Early in 1996, a study demonstrated that the interactions and combinations of sound and vision may contribute to increase the sway behavior^9^. From the above, the authors hypothesized that WGN might affect the balance performance. But the authors speculated that the effects should be studied and conducted in certain conditions, such as appropriate intensity and/or frequency.

As early as 1993, Collins and De Luca defined open-loop (related to the short-term persistence) and closed-loop (related to the long-term persistence) theories concerning the balance control during quiet upright stance. They pointed out that the short-term persistence is related to an open-loop, indicating a feedforward control that disenables the effective feedback from the sensorimotor loops; whereas, the long-term anti-persistence is associated with an closed-loop (feedback) control^10^. This theory is commonly mentioned in the balance study, both in quiet standing studies^11–15^ and dynamic standing studies^16–19^. However, this theory is controversial. Later, Peterka used a simple feedback model of upright stance. He found that this model can produce stabilogram diffusion plots which shapes are very similar to actual stabilogram diffusion functions. He therefore queried the Collins’ theory of ‘open-loop’ versus ‘closed-loop’ because the physiological stabilogram diffusion functions can be produced without postulation of a non-linear open-loop operation^20^. Detrended fluctuation analysis (DFA) is a nonlinear time series analysis^21^ commonly used when analyzing the center of pressure (COP) trajectory^22^. However, serials of works by van der Kooji er al argued that the method of direct approach is only appropriate for identification of open-loop systems. It is not applicable for the dynamics within a closed-loop system since it is impossible to make an inference on the control dynamics based on observations without perturbations^23–25^. Nevertheless, DFA was widely used in the previous studies for investigating the balance control since it is a direct and simple approach. By using DFA, recently Kodama et al. came up with the conclusion that the fast-scale persistent region captures the trembling component^16^, which is associated with the elementary spinal and muscular reflexes^26^, while the anti-persistent slow-scale region indexes the rambling dynamics^16^, which relate to CNS modulation^26^. Although it is controversial, we hypothesized that investigation of the changes of the rambling and trembling in the dynamic structure of COP trajectories might be helpful to further understand the mechanisms of the motor control under the conditions such as vibrotactile feedback training^16^ or dynamic swaying force platform. It is desired to distinguish the dynamic control from one subsystem (For example, the CNS) to the others (For example, muscle and/or tendon).

The present study aims to explore the effects of WGN of different intensities and frequencies. The authors attempt to test their hypothesis whether a particular intensity and frequency of WGN can improve the dynamic balance performance of healthy young adults. Moreover, employing DFA, the authors also want to determine which scale region can be affected by WGN of certain intensities and frequencies. The authors believe that the findings of this study will be helpful in understanding the worthiness of WGN as an adjuvant therapy in rehabilitation training to lower fall risk.

## Results

A total of 20 participants (11 males and 9 females) were enrolled in the present study. The average age of the participants was 22.55 ± 2.05 years. The average body mass was 58.58 ± 8.00 kg. The average height was 170.06 ± 8.34 cm.

The analysis of COP trajectory parameters shows that the LCT and Rx of the COP trajectories in the intensity block (analysis of different strengths of WGN) were smaller than those in the control group (Table S1). When the WGN was set at 75 dB, the values reached the minimum (LCT 175.4 ± 44.5; Ry 10.8 ± 3.0) and were significantly lower than those in the control (LCT, *df* = 119, *P* = 0.0256, *F* = 2.7557; Ry, *df* = 119, *P* = 0.0331, *F* = 2.8296); whereas, the Rx and *S* reached the minimum at 85 dB (Rx 2.9 ± 0.7; *S* 44.1 ± 24.0). Only the reduction of Rx was significant (*df* = 119, *P* = 0.0139, *F* = 1.1496) (Table 1 and Table S1). Almost all COP parameters stimulated with WGN of different frequencies were lower compared with those in the control group (Table S2). However, no significant difference was found in these data (*P* > 0.05 vs. the control group), indicating that WGN frequency did not affect the dynamic balance performance of the participants (Table 1).

**Table 1:** *P* values between the control and noise stimulation groups.

Parameters	LCT	Rx	Ry	S
***P* Value (F, df) versus Control**				
Intensity				
55 dB	0.1032 (1.2500,119)	0.9808 (1.1676,119)	0.1029 (1.3265,119)	0.5431 (0.4326,119)
65 dB	0.3839 (1.1995, 119)	0.4762 (0.7458,119)	0.4223 (1.2488,119)	0.8246 (0.6370,119)
75 dB	0.0256* **(2.7557,119)**	0..1236 (0.9528,119)	0.0331* **(2.8296,119)**	0.5225 (0.4711,119)
85 dB	0.0810 (1.6431,119)	0.0139* **(3.1495,119)**	0.1080 (1.6529,119)	0.2004 (0.9876,119)
Frequency				
500 Hz	0.6167 (0.9622,119)	0.1971 (1.0713,119)	0.8320 (1.0890,119)	0.1492 (0.6345,119)
1500 Hz	0.3086 (1.1358,159)	0.1315 (1.2122,159)	0.8236 (2.5246,159)	0.4997 (0.5445,159)
2500 Hz	0.4235 (0.9991,159)	0.1746 (1.0961,159)	0.8636 (1.9045,159)	0.4374 (0.3906,159)
3500 Hz	1.2473 (1.6431,159)	0.7421 (1.3904,159)	0.7508 (2.0323,159)	0.2437 (1.0949,159)

Data were analyzed by a one-way repeated measure analysis of variance followed by Dunnett’s post hoc correction for multiple comparisons.

df, degree of freedom; LCT, length of the COP sway trajectory; Rx, range of the COP sway trajectory in the A/P direction; Ry, range of the COP sway trajectory in the M/L direction; S, COP sway trajectory envelope area.

* means p *<* 0.05

**Figure 1:**
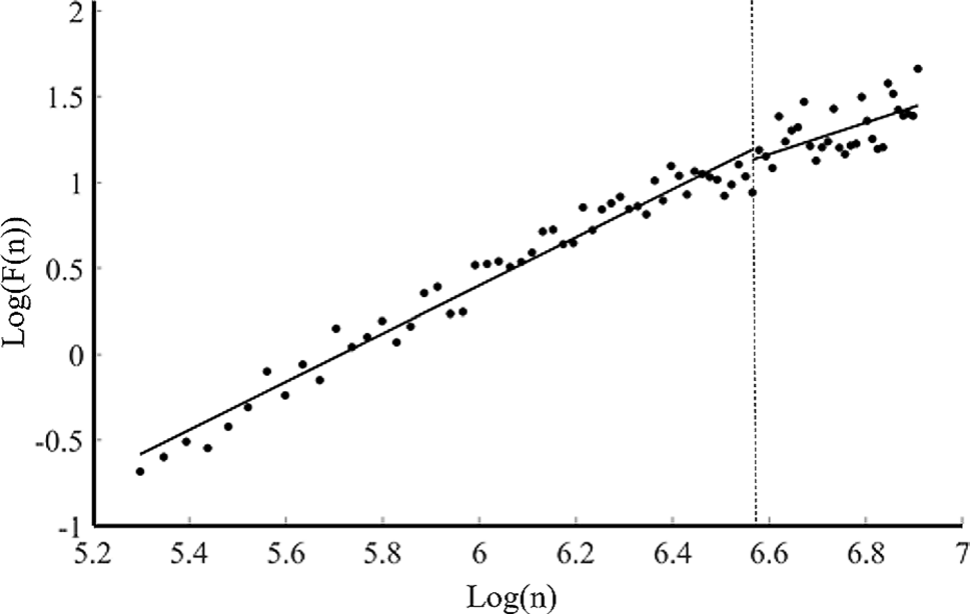
A typical log–log diffusion diagram generated by DFA of the COP data. Schematic representation of the typical log–log diffusion plots resulting from DFA of the COP data. The cross-over phenomenon was detected with slope < 1 or slope > 1. Data of this image were processed using Matlab (R2017a, https://www.mathworks.com/products/new_products/release2017a.html).

Figure 1 is a representative diagram of the log–log diffusion graph generated by DFA of the COP data. From the results of the fitting line, the whole diffusion graph can be fitted into two lines, which slopes < 1 or > 1, respectively. This indicates that the time series of the dynamic COP speed exhibited a sustained correlation in the short term and an anti-sustained correlation in the long term, which is in agreement with a CP phenomenon. These data confirmed the reliability of the experimental system of this study.

**Figure 2:**
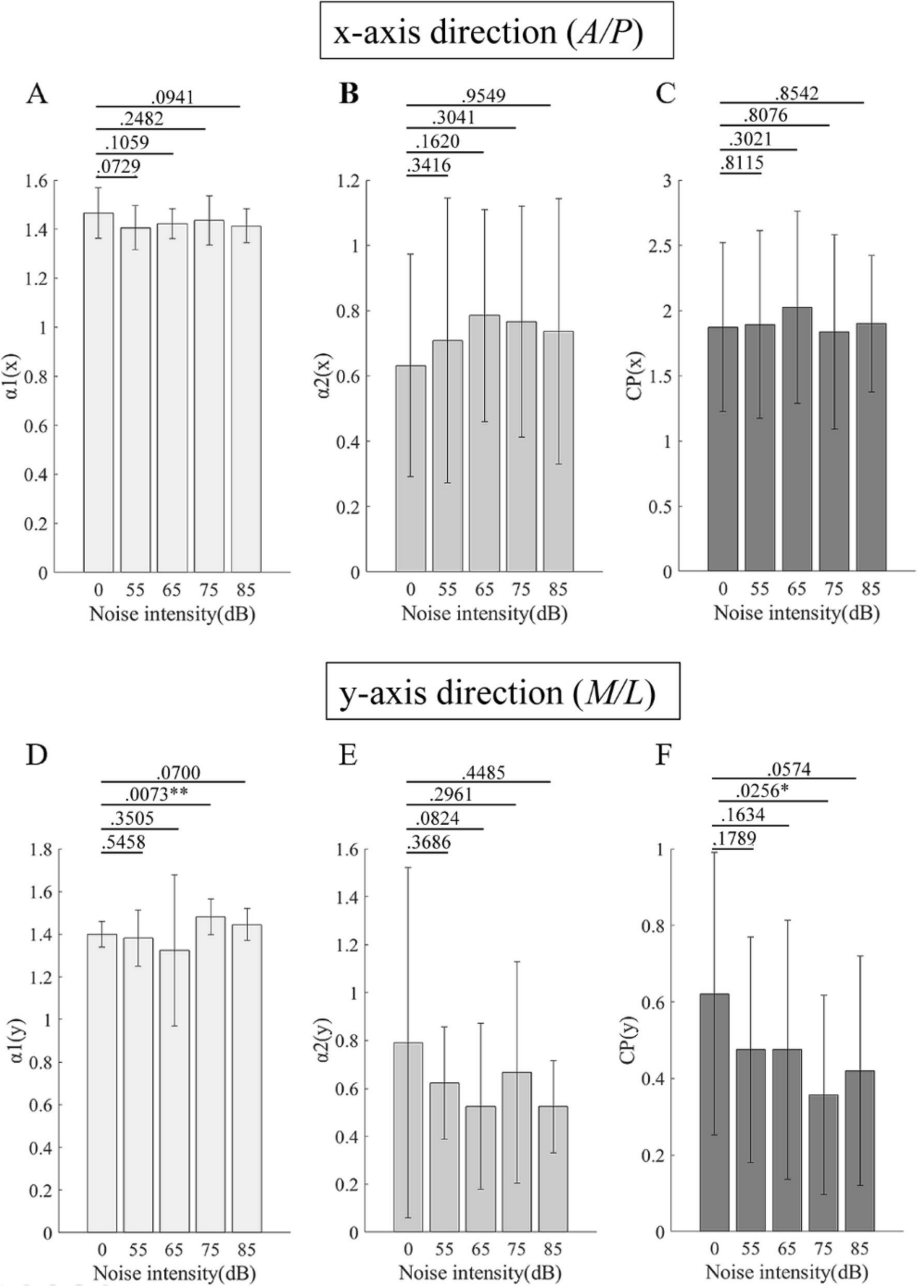
Fractal characteristics data of DFA stimulated with different WGN intensities. (**A**–**C)** are the data at the x-axis (*A/P*) direction; (**D**–**F)** are the data at the y-axis (*M/L*) direction. (**A**) Short-term scaling index. (**B**) Long-term scaling index. (**C**) The intersection point between short-term and long-term fluctuations. (**D**) Short-term scaling index. (**E**) Long-term scaling index. (**F**) The intersection point between short-term and long-term fluctuations. Data are presented as average ± standard deviation (SD); *means *P* < 0.05; **means *P* < 0.01. A/P = anterior/posterior; M/L = medial/lateral; CP = crossover point. Data of the images (A-F) were processed using Matlab (R2017a, https://www.mathworks.com/products/new_products/release2017a.html).

Figure 2 shows the fractal characteristics data of DFA (COP speed) stimulated with different WGN intensities. The values of the short-term slope (Fig. 2A), long-term slope (Fig. 2B), and CP (Fig. 2C) in the *A/P* direction did not have significant differences when stimulated with different WGN intensities (vs. control 0 dB). However, as for the data in the *M/L* direction, 75 dB WGN stimulation significantly enhanced the value of the short-term slope (*df* = 119, *P* = 0.0073, *F* = 3.5180; vs. control 0 dB) (Fig. 2D) and reduced the CP value (*df* = 119, *P* = 0.0256, *F* = 2.9782; vs. control 0 dB) (Fig. 2F).

**Figure 3:**
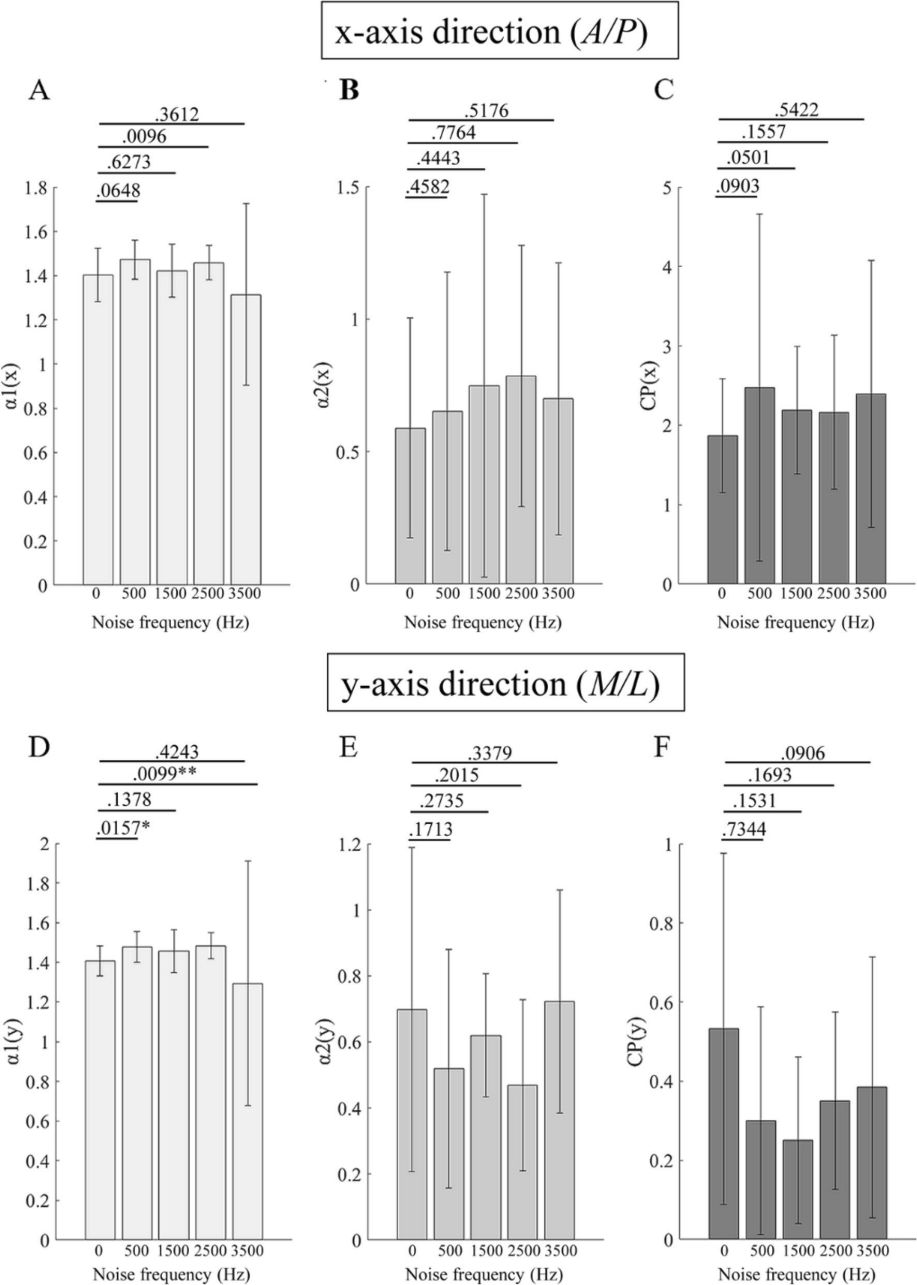
Fractal characteristics data of DFA stimulated with different WGN frequencies. (**A–C)** are the data at x-axis (*A/P*) direction; (**D–F**) are the data at the y-axis (*M/L*) direction. (**A**) Short-term scaling index. (**B**) Long-term scaling index. (**C**) The intersection point between short-term and long-term fluctuations. (**D**) Short-term scaling index. (**E**) Long-term scaling index. (**F)** The intersection point between short-term and long-term fluctuations. Data are presented as average ± standard deviation (SD); *means *P* < 0.05; **means *P* < 0.01. A/P, anterior/posterior; M/L,  medial/lateral; CP,  crossover point. Data of the images (**A**–**F**) were processed using Matlab (R2017a, https://www.mathworks.com/products/new_products/release2017a.html).

Figure 3 shows the fractal characteristics data of DFA (COP speed) stimulated with different WGN frequencies. The values of the short-term slope (Fig. 3A), long-term slope (Fig. 3B), and CP (Fig. 3C) in the *A/P* direction did not have significant differences when stimulated with different WGN intensities (vs. control 0 Hz). However, as for the data in the *M/L* direction, 500 Hz and 2500 Hz WGN stimulation significantly enhanced the values of the short-term slope (*df* = 119, P_500Hz_ = 0.0157, *F* = 2.9655; and *df* = 159, P_2500Hz_ = 0.0099, *F* = 3.3252; vs. control 0 Hz) (Fig. 3D).

## Discussion

In the present study, the authors investigated the effects of WGN of different intensities and frequencies on the dynamic balance performance in healthy young adults. The results of this study with regard the COP parameters indicated that only with 75–85 dB WGN, the COP parameters improved. WGN frequency did not affect the dynamic balance performance of the participants. The DFA results indicated that 75 dB WGN stimulation enhanced the short-term index and reduced the CP value. Stimulation with 500 and 2500 Hz WGN significantly enhanced the short-term index. These results suggest that 75 dB WGN and 500 and 2500 Hz WGN improve the dynamic balance performance. To the best of the authors’ knowledge, this is the first study investigating the effects of WGN in different conditions. The findings of this study imply that WGN can be considered as an adjuvant treatment for the rehabilitation of patients with balance impairments, which require further investigation in patients.

Although exposure to WGN of all intensities and frequencies exhibited improvement tendency in all of the COP parameters (Tables S1and S2), only 75 dB WGN significantly improved the LCT and Ry and only 85 dB WGN significantly reduced the Rx. The frequency block did not show any difference (Table 1). These results indicated that a certain intensity of WGN is indispensable to achieve a remarkable amelioration of dynamic balance impairments. The mechanisms lie in the fact that noise stimulation improves the vestibular function and makes the vestibular nerve more sensitive to body swing^27,28^. And only large enough noise intensities can cause a stochastic resonance phenomenon and thus result in balance improvements^29^. The results of this study show that the WGN intensity threshold is between 75 and 85 dB. These results are partly in accordance with the previous studies, whose results showed that the noise intensity threshold was 75 dB on static balance^8,29^.

With regard to DFA, at first, the representative diagram of the log–log diffusion graph exhibited two slopes with a CP point (Fig. 1): a short-term persistence and a long-term anti-persistence. This pattern is in agreement with the velocity-based postural control model, which is velocity-based rather than position-based^22^. These data confirmed the reliability of the experimental system of this study and the rationality of using DFA. The results of DFA in this study indicated that 75 dB WGN stimulation (*M/L*) significantly enhanced the values of the short-term slope and reduced the CP value (Fig. 2). Stimulation with 500 Hz and 2500 Hz WGN (*M/L*) significantly enhanced the values of the short-term slope (Fig. 3). These data indicate that both of the intensity and frequency affect the short-term persistence, namely, the open-loop, indicating a feedforward control that disenables the effective feedback from the sensorimotor loops, which is reportedly associated with the trembling component^16^. Since the authors have known that the trembling relates to the elementary spinal and muscular reflexes^26^, rather than the CNS reflexes. When the sway starts at a low speed, it is in the persistence model. Now, the CNS does not need to respond to keep balance, only relying on the elementary spinal and muscular reflexes (trembling). Once the speed is enhanced over the CP point, it will be changed to the anti-persistence model, which relates to a closed-loop feedback control (rambling), and the CNS will respond to keep balance. Obviously, the results of this study indicate that WGN affects only the open-loop, or trembling, control, in which balance is maintained by the elementary spinal and muscular reflexes. WGN will not influence the closed-loop control, in which the CNS starts responding. This may give a hint that WGN is appropriate for low-speed rehabilitation training (≤ 60°/s) but has no effect on the high-speed rehabilitation training (over 240°/s)^30^. This deduction required further investigation.

Here, all experiments were conducted with the participants’ eyes opened to confirm that all the sensory input is available. The platform swings in the *M/L* direction. It is reasonable that the changes of COP trajectory parameters in the *M/L* direction are larger than those in the *A/P* direction, induced by the WGN stimulation (in both COP parameter analysis and DFA). But the position of the platform slightly affects the results of this study since, here, the WGN effects were attributed to a velocity-based model.

There are several limitations in this study. (1) The present study only recruited 20 participants. The small sample size might reduce the strength of the evidence. (2) This study only performed the experiments with eyes opened without performing the conditions with eyes closed, which is extremely important in a dynamic balance research. (3) The findings of this study need to be verified in patients for elucidating the clinical value of WGN in ameliorating the dynamic balance performance. (4) This study employed a traditional DFA method for data preprocessing. However, van der Kooji pointed out that this direct approach might bring erroneous results since it is inappropriate for identifying the dynamics within a closed-loop^24^. It is impossible to distinguish the dynamics of one subsystem from the others^23^. Albeit in terms of the aim of this study, namely, exploring the effects of WGN of different intensities and frequencies on dynamic balance performance, this concern did not influence the results/conclusion of the present study, for obtaining more compelling evidence, the authors will employ approaches such as indirect approach of joint input–output, which introduced in the van der Kooji’s study^24^ for the further investigation of these issues in the future study. (5) The corrections of the COP measurements were not performed. Although the potential influence of inertia of the force-plate and the feet were under the same conditions in all experiments, and the data-preprocessing may counteract this contribution, thereby did not affect the results and conclusion of this study, it might give a rather big bias to the reported COP values. This issue will be addressed in the future investigation.

## Conclusions

This study verified that certain strengths of WGN (75–85 dB in this study) rather than the frequency significantly improved the dynamic balance performance of healthy young adults. The DFA results imply that WGN mainly affected the short-term persistence, which is associated with open-loop, trembling component, and the elementary spinal along with muscular reflexes. These results indicate the potential of WGN to be considered as an adjuvant therapy for low-speed gesture training, which requires further investigation.

## Methods

### Participants

A total 20 healthy participants were recruited in this study. Participants who were healthy, aged < 30 years old, with body mass index (BMI) of < 24.7, without history of postural or vestibular deficits, and compliant were included in the study. Those who were with neurological diseases and other diseases, and with BMI of > 25 and could not finish the experiments were excluded. This study was designed and conducted complying with the guidelines of the 2000 revision of the Declaration of Helsinki of the World Medical Association and was approved and supervised by the ethical committee of Shanghai Ruijin Hospital Luwan Branch (Approval No: LWEC2019017). All participants provided signed informed consents after being introduced to whole experimental protocol in detail. Written informed consent was obtained from the individuals for the publication of any potentially identifiable images or data included in this article.

### Noise stimulation

The authors employed a noise generation module compiled by the MATLAB platform (R2017a, The MathWorks Inc., Natick, MA, USA) to generate WGN. The noise signals were sent to the participants through headsets (Beoplay H9i, Bang & Olufsen, Struer, Denmark). The intensity and frequency of WGN could be adjusted by setting specific parameters on the computer module. The high-precision digital noise meter PM6708 (Shenzhen Huayi Peakmeter Technology Co., Ltd., Shenzhen, China) was used to perform noise detection on the test environment. A 10 s noise-level test was performed before the experiment to ensure that the test was conducted in a quiet environment (less than 30 dB).

### Dynamic balance measurement

**Figure 4:**
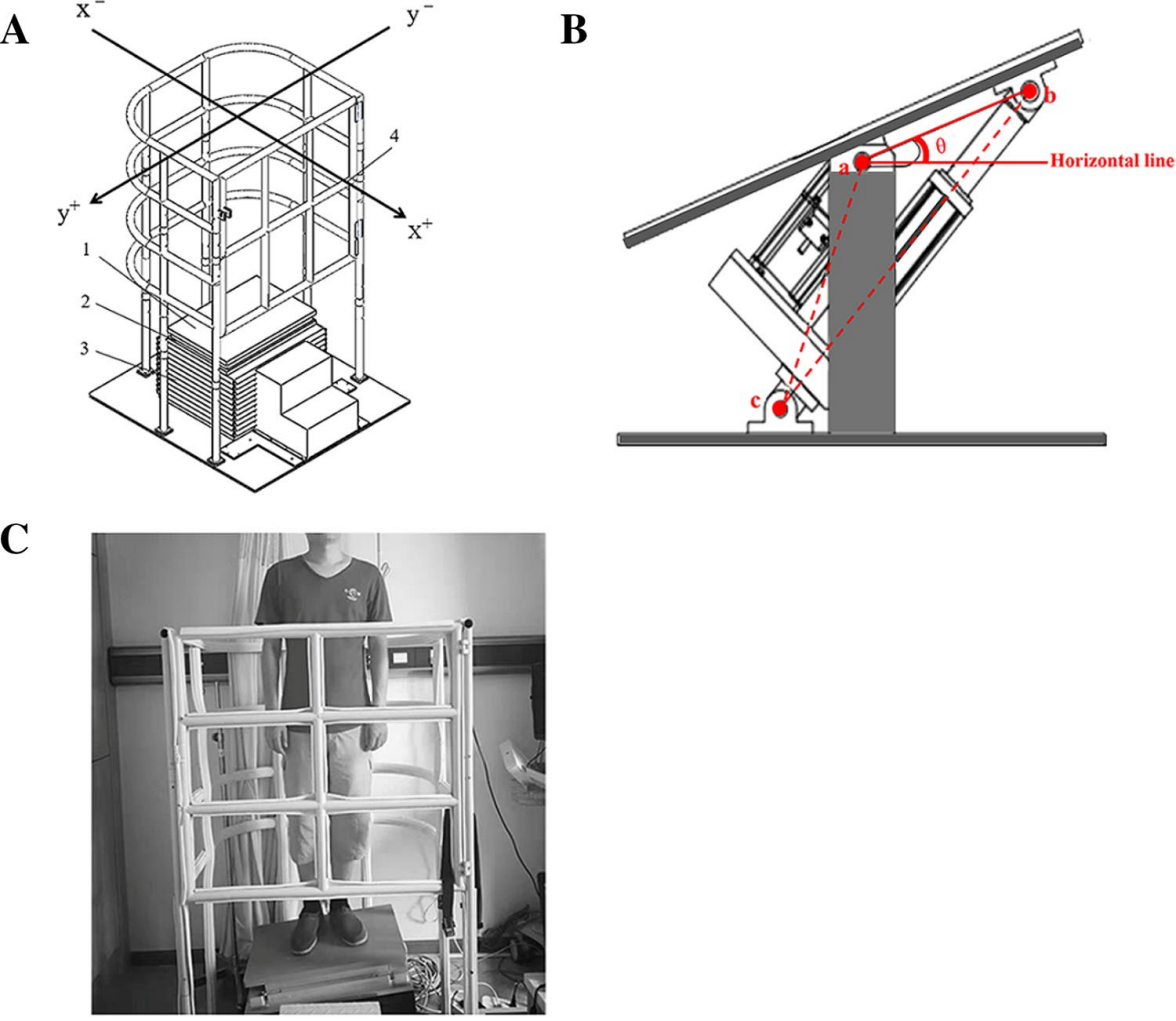
Diagram of the dynamic balance force platform (DBFP). (**A**) The mechanical structure of the DBFP (the x-axis and y-axis directions are shown in the Figure.) The x-axis corresponds to the anterior/posterior (*A/P*) direction of the human body; the y-axis corresponds to the medial/lateral (*M/L*) direction. During the test, the participants faced the x + direction. (**B**) Internal structure of the swing mechanism assembly, θ represents the rotation angle. θ represents the rotation angle between the flat plate and the horizontal line, which adjusts the amplitude of sway. (**C**) A representative image during the experiment. Tests were performed with eyes opened.

A self-made dynamic balance force platform was developed to assess the COP coordinates under a swaying condition, according to a previous study^31^. Detailed information concerning the assessments is shown in Fig. 4. The platform consists of the following: (1) a precise force plate (BP400600) (Advanced Mechanical Technology Inc., MA, USA), (2) a swaying plate (600 mm [width] × 400 mm [length]), (3) a swaying mechanism assembly, and (4) a safety ring. The platform was set up as previously described^31^. Briefly, main parameters of this platform are sway frequency and amplitude. Sway amplitude is adjusted by the angle θ, which is a sharp angle between the flat plate and the horizontal line. It can be controlled by the servo motor motion control system (Fig. 4). The control pattern is computer + servo motor + force platform. Computer sends signals to the servo drive to run the motor and then control the parameters of the force platform (angle θ and frequency of sway). Once the rotation angle θ is set up, the COP coordinates can be measured through multiplication of a transformation matrix^31^. During the test, the swaying plate swings along the x-axis with an amplitude of ± 4° and frequency of 1 Hz. The sampling frequency for the pressure transducer was 500 Hz. Data were low-pass filtered with a zero-lag, fourth-order Butterworth filter with a cut-off frequency of 8 Hz.

### Experimental protocol

**Figure 5:**
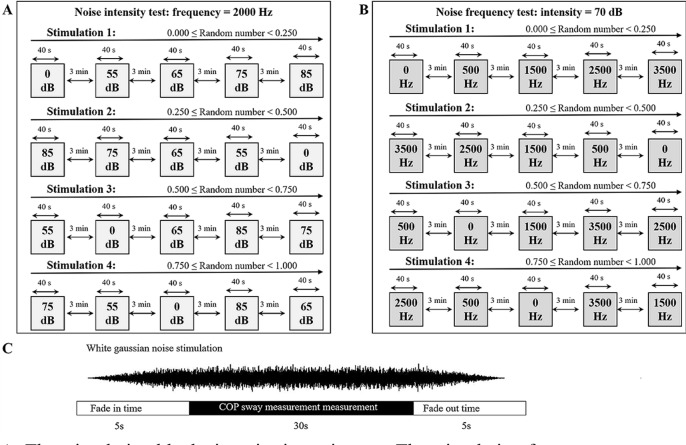
Diagram of the experimental protocol. (**A**) The stimulation blocks in noise intensity test; The stimulation frequency was set to 2000 Hz. Four stimulation blocks with different intensity sequence were previously defined. The stimulation block undergone by a participant was determined by the random number he/she obtained. (**B**) The stimulation blocks in noise frequency test; The stimulation intensity was set to 70 dB. Four stimulation blocks with different frequency sequence were previously defined. The stimulation block undergone by a participant was determined by the random number he/she obtained. (**C**) Data acquisition procedure.

The experimental protocol is shown in Fig. 5. The participants were asked to comfortably stand on the force plate with their feet kept shoulder-width apart (300 mm width of the stance) and their eyes focused on the mark 2.5 m away in front. The test period was set to 40 s for each state, with the first 5 s allocated for the fade-in, the next 30 s for the formal test, and the last 5 s for the fade-out. Data for the first and last 5 s were removed to avoid any disturbing effects at the beginning and ending. A total of five dynamic COP measurements in each block (intensity block and frequency block) were performed for each participant. The measurements in each block were staggered to avoid potential interference. During the intensity experiment, the WGN frequency was set to 2000 Hz^31^, and the WGN intensity was changed from 0 to 55 dB, 65 dB, 75 dB, and 85 dB; 0 dB (no stimulus) was set as the control. In the frequency experiment, the WGN intensity was set to 70 dB^31^, and the WGN frequency was changed from 500 to 1500 Hz, 2500 Hz, and 3500 Hz. Stimulation at 0 Hz (no stimulus) was set as the control. The interval between each condition was 3 min. In order to avoid possible bias caused by the sequence of intensity and frequency, the present study employed a simple randomization method. A random number (0.000–1.000) was generated by a scientific calculator (F605G, Canon, Tokyo, Japan). As shown in Fig. 5, four stimulation blocks with different parameter sequence were previously defined for both tests. Stimulation block for a participant was determined by the random number he/she obtained. And the stimulation sequence was blinded to the participant (Fig. 5).

### Data preprocessing

#### STEP 1. Analysis of the COP parameters with a traditional method

The MATLAB software (R2017a, The MathWorks Inc., Natick, MA, USA) was used to analyze the experimental data.

Because the starting point of the COP varies in each measurement, the mean of the obtained displacement data was regarded as the center of the coordinates. In the authors’ calculations, *x* represents the *x*-axis (*A/P*) direction, and *y* represents the *y*-axis (*M/L*) direction. The coordinate equation is as follows:

1 $$ x = x^{\prime} - \overline{x} $$

2 $$ y = y^{\prime} - \overline{y} $$

where $x$ and *y* are the displacements in the *A/P* and *M/L* directions with the offset removed, *x′*_and *y′*_ are the raw data of the displacement in the *A/P* and *M/L* directions, *x* and *y* are the offsets in the *A/P* and *M/L* directions.

In this study, the participant’s balancing ability was assessed by measuring the length of the COP sway trajectory (represented as *LCT*), the mean range of the COP sway trajectory in the *A/P* and *M/L* directions (represented as *Rx* and *Ry*), and COP sway trajectory envelope area (represented as *S*). The corresponding equations are as follows:

3 $$ LCT = \sum\limits_{i = 1}^{n} {\sqrt {(x_{i + 1} - x_{i} )^{2} + (y_{i + 1} - y_{i} )^{2} } } $$

4 $$ Rx = \frac{1}{n}\sum\limits_{i = 1}^{n} {\sqrt {(x_{i + 1} - x_{i} )^{2} } } $$

5 $$ Ry = \frac{1}{n}\sum\limits_{i = 1}^{n} {\sqrt {(y_{i + 1} - y_{i} )^{2} } } $$

where *n* is the number of data samples and the subscript *i* represents certain one data. *S* is calculated through the following procedure: firstly, the outer contour points of the COP trajectory were searched, which constituted a convex polygon containing all COP data, and then the area of the convex polygon was calculated as the envelope area of the COP trajectory.

#### STEP 2. Analysis of the COP parameters with a DFA method

The COP data collected by the volunteers were further analyzed through DFA, and the classification of COP was studied by the scale index method. The calculation steps of DFA are as follows:

- *Step 1* Calculation of the cumulative deviation of time series [*v*(i), *t* = 1,2,…,N] of the COP swing velocity (*v*). Δ*t* is 0.002 s and the calculation formula of *v* is (6) as follows:
  6 $$ v = \sqrt {(x_{i + 1} - x_{i} )^{2} + (y_{i + 1} - y_{i} )^{2} } /\Delta t $$
  7 $$ k(i) = \sum\limits_{i = 1}^{n} {[v(i) - \overline{v}]} $$

where *k(i)* is the sequence reconstruction, which was divided into segments with an equal interval of *q*, and *q* is called as the scale of interval. The least squares method was adopted to carry out second-order polynomial fittings for each segment, denoted as *k*_*q*_*(i)*.

- *Step 2* Calculation of the fluctuation (root mean square) of the cumulative time series as follows:
  8 $$ F(q) = \sqrt {\frac{1}{n}\sum\limits_{i = 1}^{n} {(k(i) - k_{q} (i))^{2} } } $$

Since *v*(*i*) was a time series with self-similarity characteristics, there was a power law relationship between the *F(q)* and *q*, which could be expressed as the following formula:

9 $$ F(q) \propto q^{\alpha } $$

where *α* is the scale index. When *α* > 1 (or *α* < 1), it shows that the time series contains information related to long range (or short range).

The authors calculated and counted *α*_*1*_ (short-term scaling exponent) and *α*_*2*_ (long-term scaling exponent) and their *crossover point (CP)*.

### Statistical analyses

Finally, the fractal characteristics data of DFA were analyzed by SPSS software (V19.00 IBM, IL, USA). All the experiments were repeated independently at least three times and the means were calculated and submitted for the multiple comparison. The normality of data distributions was analyzed using the Kolmogorov–Smirnov test. The values of Kolmogorov–Smirnov test were over 0.05. Hence the data were regarded as normally distributed. Data were then analyzed by a one-way repeated measure analysis of variance (ANOVA) Followed by Dunnett’s post hoc correction for multiple comparisons. Individual parameters were tested in separate ANOVAs for avoiding potential biases^32^.

The data were presented as mean ± standard deviation (SD). The level of significance was set at *P* < 0.05 for all statistical analyses.

## Supplementary Information

Supplementary Information

## Data Availability

The datasets used and/or analyzed during the current study are available from the corresponding author on reasonable request.
