# Supplementary material for: Effects of white Gaussian noise on dynamic balance in healthy young adults
Source: Sci Rep. 2021 Mar 9;11:5455. doi: 10.1038/s41598-021-84706-8 (PMC7943824; doi:10.1038/s41598-021-84706-8)
Supplement: Supplementary file 1 — Supplementary Information [file 41598_2021_84706_MOESM1_ESM.docx]

**Effects of white Gaussian noise on dynamic balance in healthy young adults**

Ziyou Zhou^1^, Can Wu^1^, Zhen Hu^2^, Yujuan Chai^5^, Kai Chen^1*^, Tetsuya Asakawa ^3,4*^

1. School of Mechanical Engineering, Hangzhou Dianzi University, Hangzhou, Zhejiang 310018, China

2. Department of Neurology, Ruijin Hospital Affiliated to Shanghai Jiao Tong University, Shanghai 200000, China

3. Department of Neurosurgery, Hamamatsu University School of Medicine, Handayama, Hamamatsu-city, Shizuoka, Japan

4. Research Base of Traditional Chinese Medicine Syndrome, Fujian University of Traditional Chinese Medicine, Fuzhou 350122, China

5. Health Science Center, School of Medical Engineering, Shenzhen University, Shenzhen 518060, China

ORCID: TA: 0000-0002-2300-3509

**Corresponding Authors:**

Kai Chen, Ph.D.

Department of Mechanical Engineering, Hangzhou Dianzi University, Hangzhou Zhejiang, China

No.1158, Xiasha 2nd Street, Jianggan District, Hangzhou, Zhejiang, China

Tel: +86-138-6815-0662; Fax: +86-0571-86919054

Email: kaicn@hotmail.com

Or

Tetsuya Asakawa, M.D., Ph.D.

Department of Neurosurgery, Hamamatsu University School of Medicine

Handayama, 1-20-1, Higashi-ku, Hamamatsu-city,

Shizuoka, 431-3192, Japan

Tel: + 81-53-435-2283; Fax: + 81-53-435-2282

E-mail: [asakawat1971@gmail.com](mailto:asakawat1971@gmail.com)

**Supplementary materials**

**Table S1. The statistical characteristics of the data between the control and noise stimulation groups in the intensity block**

|  | *Mean* | *SD* | *CI* | *Min* | *Max* |
| --- | --- | --- | --- | --- | --- |
| (a) *LCT* |  |  |  |  |  |
| Control | 197.6 | 16.9 | 170.2 － 225.1 | 108.9 | 303.7 |
| 55 dB | 182.7 | 51.1 | 158.2 － 207.2 | 115.1 | 309.1 |
| 65 dB | 186.6 | 52.1 | 161.6 － 211.6 | 108.0 | 314.5 |
| 75 dB | 175.4 | 44.5 | 154.1 － 196.8 | 109.6 | 276.3 |
| 85 dB | 176.0 | 34.4 | 159.5 － 192.5 | 121.2 | 230.8 |
| (b) *Rx* |  |  |  |  |  |
| Control | 3.1 | 0.6 | 2.9 － 3.5 | 2.2 | 4.8 |
| 55 dB | 3.2 | 0.6 | 2.9 － 3.5 | 2.4 | 4.7 |
| 65 dB | 3.1 | 0.8 | 2.7 － 3.5 | 1.4 | 5.5 |
| 75 dB | 3.0 | 0.7 | 2.6 － 3.3 | 2.1 | 5.2 |
| 85 dB | 2.9 | 0.7 | 2.6 － 3.2 | 1.9 | 4.7 |
| (c) *Ry* |  |  |  |  |  |
| Control | 12.2 | 3.9 | 10.3 － 14.1 | 6.1 | 19.7 |
| 55 dB | 11.2 | 3.4 | 9.6 － 12.8 | 6.5 | 19.8 |
| 65 dB | 11.5 | 3.5 | 9.9 － 13.2 | 6.4 | 20.1 |
| 75 dB | 10.8 | 3.0 | 9.3 － 12.3 | 5.7 | 16.8 |
| 85 dB | 10.9 | 2.3 | 9.8 － 12.0 | 7.0 | 14.5 |
| (d) *S* |  |  |  |  |  |
| Control | 52.0 | 23.9 | 40.5 － 63.5 | 17.1 | 106.2 |
| 55 dB | 55.8 | 36.3 | 38.4 － 73.2 | 17.5 | 171.4 |
| 65 dB | 53.2 | 29.9 | 38.8 － 67.57 | 16.6 | 132.1 |
| 75 dB | 48.1 | 34.8 | 31.4 － 64.8 | 18.7 | 170.7 |
| 85 dB | 44.1 | 24.0 | 32.5 － 55.6 | 18.9 | 123.6 |

LCT = length of the COP sway trajectory; Rx = range of the COP sway trajectory in the A/P direction; Ry = range of the COP sway trajectory in the M/L direction; S = COP sway trajectory envelope area; CI = confidence interval; SD = standard deviation; Min = minimum; Max = maximum

**Table S2. The statistical characteristics of the data between the control and noise stimulation groups in the frequency block**

|  | *Mean* | *SD* | *CI* | *Min* | *Max* |
| --- | --- | --- | --- | --- | --- |
| (a) *LCT* |  |  |  |  |  |
| Control | 203.2 | 49.5 | 179.4 － 227.0 | 125.1 | 336.5 |
| 500 Hz | 198.7 | 50.5 | 174.5 － 223.0 | 131.5 | 328.8 |
| 1500 Hz | 194.9 | 46.5 | 172.6 － 217.2 | 135.0 | 305.5 |
| 2500 Hz | 197.8 | 49.6 | 174.0 － 221.6 | 143.4 | 314.9 |
| 3500 Hz | 198.8 | 44.4 | 177.5 － 220.1 | 110.8 | 283.4 |
| (b) *Rx* |  |  |  |  |  |
| Control | 3.4 | 1.8 | 2.5 － 4.3 | 2.3 | 11.1 |
| 500 Hz | 2.8 | 0.5 | 2.5 － 3.1 | 2.0 | 3.9 |
| 1500 Hz | 2.8 | 0.4 | 2.6 － 2.9 | 2.2 | 3.3 |
| 2500 Hz | 2.8 | 0.4 | 2.6 － 3.0 | 2.1 | 3.8 |
| 3500 Hz | 3.2 | 1.3 | 2.6 － 3.8 | 2.1 | 8.4 |
| (c) *Ry* |  |  |  |  |  |
| Control | 12.4 | 3.5 | 10.7 － 14.1 | 7.6 | 21.7 |
| 500 Hz | 12.5 | 3.4 | 10.9 － 14.1 | 8.1 | 21.4 |
| 1500 Hz | 12.3 | 3.2 | 10.8 － 13.8 | 8.3 | 19.7 |
| 2500 Hz | 12.5 | 3.3 | 10.9 － 14.1 | 8.9 | 20.6 |
| 3500 Hz | 12.2 | 3.0 | 10.8 － 13.7 | 6.7 | 17.9 |
| (d) *S* |  |  |  |  |  |
| Control | 47.4 | 13.9 | 40.7 － 54.1 | 25.9 | 81.4 |
| 500 Hz | 41.8 | 17.5 | 33.4 － 50.2 | 18.6 | 81.6 |
| 1500 Hz | 45.0 | 18.9 | 35.9 － 54.0 | 18.6 | 90.7 |
| 2500 Hz | 44.4 | 22.3 | 33.7 － 55.1 | 17.0 | 103.9 |
| 3500 Hz | 43.1 | 13.3 | 36.7 － 49.5 | 20.6 | 68.1 |

LCT = length of the COP sway trajectory; Rx = range of the COP sway trajectory in the A/P direction; Ry = range of the COP sway trajectory in the M/L direction; S = COP sway trajectory envelope area; CI = confidence interval; SD = standard deviation; Min = minimum; Max = maximum
